# Innovative approaches in QSPR modelling using topological indices for the development of cancer treatments

**DOI:** 10.1371/journal.pone.0317507

**Published:** 2025-02-21

**Authors:** Xiaolong Shi, Saeed Kosari, Masoud Ghods, Negar Kheirkhahan

**Affiliations:** 1 Institute of Computing Science and Technology, Guangzhou University, Guangzhou, China; 2 Department of Applied Mathematics, Semnan University, Semnan, Iran; Federal University of ABC, BRAZIL

## Abstract

This paper provides a comprehensive review of quantitative structure-property relationships (QSPR) about to cancer drugs, with a focus on the application of topological indices (TI) and data analysis techniques. Cancer is a serious and life-threatening disease for which no complete cure currently exists. Consequently, extensive research is ongoing to develop new therapeutic agents. The application of topological indices in chemistry and medicine, particularly in the investigation of the molecular, pharmacological, and therapeutic properties of drugs, has become a significant tool. This article investigates the potential of Temperature indices in analyzing the physicochemical properties of drugs used for cancer treatment. The approach employs QSPR modeling to establish correlations between the molecular structure of a compound and its physical and chemical properties. The analysis covers a range of Cancer drugs, including Aminopterin, Convolutamide A, Convolutamydine A, Daunorubicin, Minocycline, Podophyllotoxin, Caulibugulone E, Perfragilin A, Melatonin, Tambjamine K, Amathaspiramide E, and Aspidostomide E. The findings demonstrate that optimal regression models (Fifty-eight models) incorporating TI can effectively predict physicochemical properties, such as Boiling Point (BP), Enthalpy (EN), Flash Point (FP), Molar Refractivity (MR), Polar Surface Area (PSA), Surface Tension (ST), Molecular Volume (MV), and Complexity (COM). This research suggests that temperature-based topological indices (TI) are promising tools for the development and optimization of cancer drugs, as demonstrated by statistically significant results with a p-value less than 0.05. In addition to the linear regression model, which performed the best, two other machine learning models, namely SVR and Random Forest, were also used for further analysis and comparison of their performance in predicting the physicochemical properties of drugs, to assess the advantages and disadvantages of each model.

## 1 Introduction

In the treatment of this disease within the human body, alkylating agents and metabolites are commonly employed. Although significant attention is devoted to the development and research of initial cancer therapies, the process of drug discovery, from identifying novel chemical compounds to obtaining regulatory approval, remains complex, costly, and time-intensive. Traditional approaches frequently encounter obstacles in compound synthesis and biological screening, leading the scientific community to explore more efficient methods for compound discovery. Chemical graph theory, an interdisciplinary field, is utilized to examine molecular structures and to establish correlations between activities, properties, and various phenomena. In this context, a molecular graph represents the structural formula of a chemical compound, with vertices corresponding to atoms and edges to chemical bonds. Chemical graph theory provides innovative tools for analyzing chemical structures, including topological indices, which serve as descriptors for the structure and specific properties of molecular graphs, typically represented as real numbers [1, 2]. Numerous studies have applied topological indices in the analysis of molecular graphs and drug structures [3–8]. A fundamental approach to exploring the relationship between a substance’s physicochemical properties and its topological indices is through Quantitative Structure-Property Relationship (QSPR) models. These models use regression analysis to examine the correlations between physical and chemical properties and topological indices. Additionally, many studies in Quantitative Structure-Activity Relationship (QSAR) have applied topological indices to drug structures [9, 10]. In this article, various temperature-based indices are evaluated across several Cancer drugs, enabling researchers to identify the associated physical properties and chemical reactions. Furthermore, in addition to linear regression, we employed Support Vector Regression (SVR) and Random Forest models to explore and assess the predictive capabilities of these methods in determining the physicochemical properties of cancer drugs. These models were applied to identify the most effective model for predicting the properties of the drugs. The results of our analysis help in selecting the best predictive model, which is crucial for improving drug design and optimizing the therapeutic effectiveness of cancer treatments [11, 12].

In this study, the drug’s structure is modeled as a graph where each vertex V(G) represents an atom and each edge E(G) signifies a chemical bond between atoms. The graphs considered are simple and connected. The degree of a vertex, defined as the number of edges incident to it, characterizes its connectivity [13].

## 2 Methodology and analysis

In this study, cancer drugs are modeled as simple graphs. To calculate the topological indices of these drug structures, we utilize techniques such as vertex partitioning, edge partitioning, and various computational methods. Our analysis is restricted to finite, simple, connected graphs. Let G denote a graph with a vertex set V and an edge set E. The degree d_u_ of a vertex u is defined as the number of vertices adjacent to u. Below is a list of the topological formulas used in this study.

**Definition 2.1** Fajtlowicz defined the concept of vertex temperature u for a connected graph G as follows [14]:

$$T_{u}=\frac{d_{u}}{n-d_{u}}$$ (1)

**Definition 2.2 **Product connectivity temperature index [15] is

$$PT(G)=\underset{uv\in E(G)}{\sum }\frac{1}{\sqrt{T_{u}\times T_{v}}}$$ (2)

**Definition 2.3** Harmonic temperature index [3] is

$$HT(G)=\underset{uv\in E(G)}{\sum }\frac{2}{T_{u}+T_{v}}$$ (3)

**Definition 2.4 **Symmetric division temperature index [16] is

$$SDT(G)=\underset{uv\in E(G)}{\sum }(\frac{T_{u}}{T_{v}}+\frac{T_{v}}{T_{u}})$$ (4)

**Definition 2.5 **Modified third temperature index [17] is

Tm3(G)=∑uv∈E(G)1(Tu+Tv) (5)

**Definition 2.6** Modified second temperature index [17] is

Tm2(G)=∑uv∈E(G)1(Tu.Tv) (6)

**Definition 2.7** Second hyper temperature indices [2] is

$$HT_{2}(G)=\underset{uv\in E(G)}{\sum }{\left(T_{u}\times T_{v}\right)}^{2}$$ (7)

**Definition 2.8** Sum connectivity temperature index [16] is

$$ST(G)=\underset{uv\in E(G)}{\sum }\frac{1}{\sqrt{T_{u}+T_{v}}}$$ (8)

**Definition 2.9** F-temperature index [16] is

$$FT(G)=\underset{uv\in E(G)}{\sum }(T_{u}^{2}+T_{v}^{2})$$ (9)

**Definition 2.10** Second temperature index [16] is

$$T_{2}(G)=\underset{uv\in E(G)}{\sum }(T_{u}\times T_{v})$$ (10)

**Definition 2.11** Reciprocal product connectivity index [16] is

$$RPT(G)=\underset{uv\in E(G)}{\sum }\sqrt{\left(T_{u}\times T_{v}\right)}$$ (11)

**Definition 2.12** First hyper temperature indices [2] is

$$HT_{1}(G)=\underset{uv\in E(G)}{\sum }{\left(T_{u}+T_{v}\right)}^{2}$$ (12)

A list of abbreviations used in the article is given in Table 1.

**Table 1:** Abbreviations list.

Meaning	Abbreviation
Boiling point	BP
Enthalpy of Vaporization	EN
Flash Point	FP
Molar Refractivity	MR
Polar Surface Area	PSA
Surface Tension	ST
Molar Volume	MV
Complexity	COM
Number of samples used for building the regression equation	N
Correlation coefficient	R
Standard error of regression coefficient	SE
Fisher’s statistic	F
Topological indices	TI
Quantitative structure-property relationship	QSPR

https://doi.org/10.6084/m9.figshare.26984122.v1

In recent years, scientists have increasingly utilized the QSPR/QSAR methodology to predict the physicochemical properties of chemical compounds through topological indices. This approach has been extensively applied in numerous studies to analyze a diverse array of drugs, including highly resistant anticancer agents, anti-COVID-19 drugs targeting the Omicron variant, breast cancer therapies, entropy tests involving benzene derivatives, nanotubes, Lyme disease treatments, and research on temperature indicators [18–22].

##  3 Mathematical computations of topological indices

This section presents the topological indices (TI) of cancer drugs and the QSPR modeling of their molecular structures.

### 3.1 Topological Index computation

Let A be a graph representing Aspidostomide E, where the edges are partitioned into distinct subsets based on specific criteria.


$$\begin{array}{c} E_{1}=\{uv\in E(A)|T_{u}=\frac{1}{24},T_{v}=\frac{2}{23}\},\;E_{2}=\{uv\in E(A)|T_{u}=\frac{1}{24},T_{v}=\frac{3}{22}\},E_{3}=\{uv\in E(A)|T_{u}=\frac{2}{23},T_{v}=\frac{2}{23}\},\\ E_{4}=\{uv\in E(A)|T_{u}=\frac{2}{23},T_{v}=\frac{3}{22}\},E_{5}=\{uv\in E(A)|T_{u}=\frac{3}{22},T_{v}=\frac{3}{22}\}. \end{array}$$

The study of the edges in A is shown in Table 2.

**Table 2:** Dividing the edges of graph A.

(Tu,Tv)\uv∈E(A)	$\left(\frac{1}{24},\frac{2}{23}\right)$	$\left(\frac{1}{24},\frac{3}{22}\right)$	$\left(\frac{2}{23},\frac{2}{23}\right)$	$\left(\frac{2}{23},\frac{3}{22}\right)$	$\left(\frac{3}{22},\frac{3}{22}\right)$
**Number of edges**	1	5	3	7	12

https://doi.org/10.6084/m9.figshare.26984170.v1

By applying Definitions 2.1 through 2.12, we obtain the following results:

1.PT(A)=∑uv∈E(A)1Tu.Tv=(1124.223)+5(1124.322)+3(1223.223)+7(1223.322)+12(1322.322)=269.732.HT(A)=∑uv∈E(A)2Tu+Tv=(2124+223)+5(2124+322)+3(2223+223)+7(2223+322)+12(2322+322)=256.913.SDT(A)=∑uv∈E(A)(TuTv+TvTu)=(124223+223124)+5(124322+322124)+3(223223+223223)+7(223322+322223)+12(322322+322322)=65.89904.Tm3(A)=∑uv∈E(A)1(Tu+Tv)=(1124+223)+5(1124+322)+3(1223+223)+7(1223+322)+12(1322+322)=128.45505.Tm2(A)=∑uv∈E(A)1(Tu.Tv)=(1124.223)+5(1124.322)+3(1223.223)+7(1223.322)+12(1322.322)=2788.41706.HT2(A)=∑uv∈E(A)(Tu×Tv)2=(124.223)2+5(124.322)2+3(223.223)2+7(223.322)2+12(322.322)2=0.00557.FT(A)=∑uv∈E(A)(Tu2+Tv2)=((124)2+(223)2)+5((124)2+(322)2)+3((223)2+(223)2)+7((223)2+(222)2)+12((322)2+(322)2)=0.78578.T2(A)=∑uv∈E(A)(Tu×Tv)=(124.223)+5(124.322)+3(223.223)+7(223.322)+12(322.322)=0.36099.RPT(A)=∑uv∈E(A)(Tu×Tv)=124.223+5124.322+3223.223+7223.322+12322.322=3.096610.HT1(A)=∑uv∈E(A)(Tu+Tv)2=(124+223)2+5(124+322)2+3(223+223)2+7(223+322)2+12(322+322)2=1.507411.ST(A)=∑uv∈E(A)1Tu+Tv=(1124+223)+5(1124+322)+3(1223+223)+7(1223+322)+12(1322+322)=59.6230

**Fig 1:**
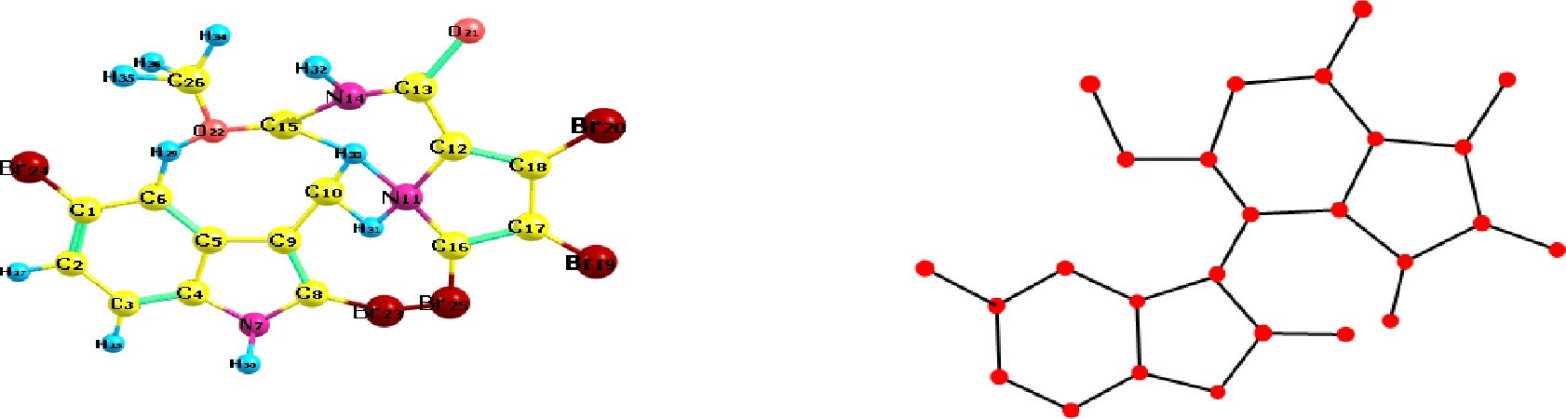
Chemical structure and Molecular graph of Aspidostomide E. a) Chemical structure of Aspidostomide E. b) Molecular graph of Aspidostomide E. https://doi.org/10.6084/m9.figshare.26984881.v1.

Fig 1 shows the Chemical structure and Molecular graph of Aspidostomide E.

**Fig 2:**
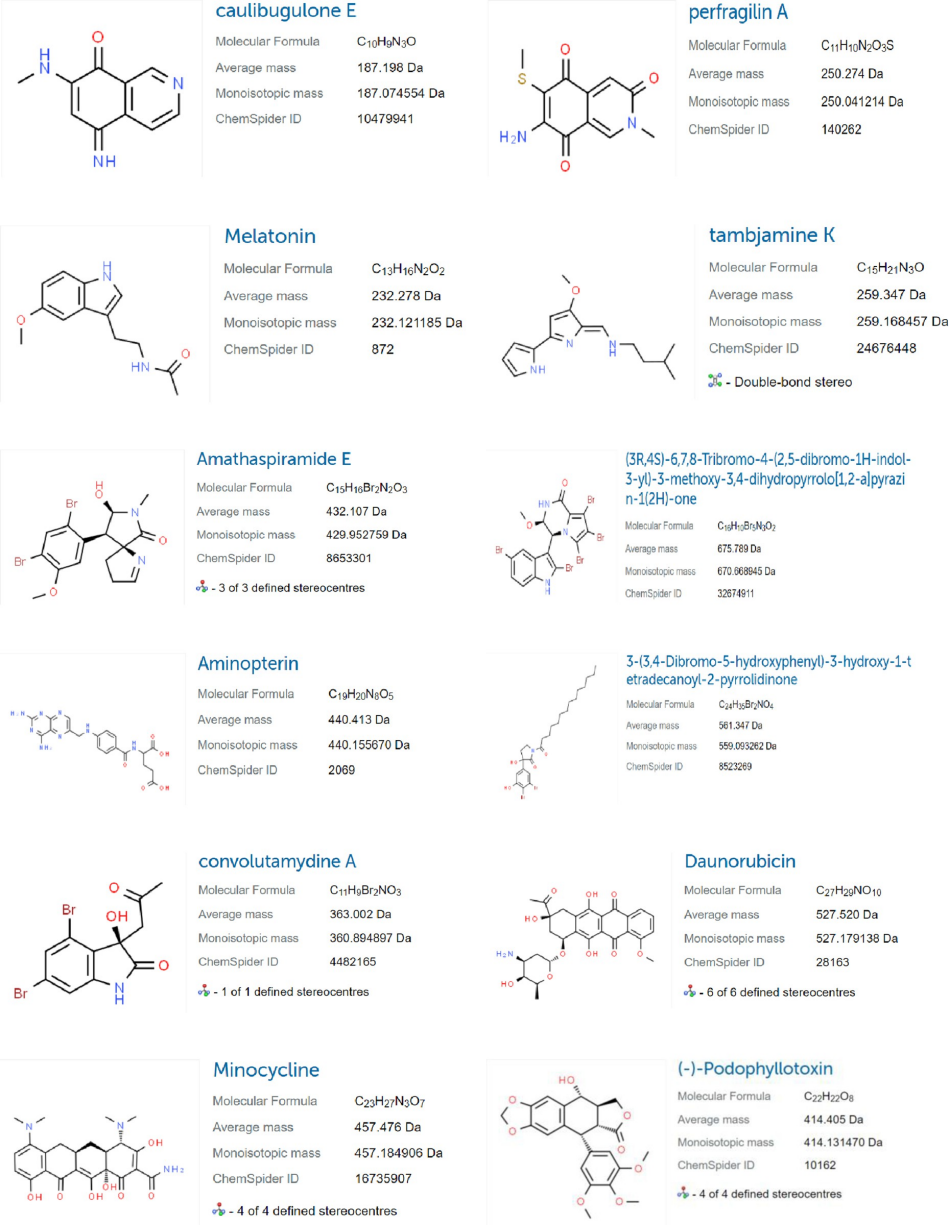
Chemical structure of cancer drugs from ChemSpider. https://doi.org/10.6084/m9.figshare.26984215.v1.

Topological indices for other drugs can be computed using the methods described in Eqs (1) to (12) from Section 2. The indices are detailed in Tables 3, 4, and Fig 2 illustrates the drugs. Additional information about these drugs can be accessed on Chemical book [23], and Table 5 summarizes their physical and chemical properties [15, 24].

**Table 3:** The values of the temperature indices of the drugs.

Drugs	PT(G)	HT(G)	FT(G)	^m^T_3_(G)	^m^T_2_(G)	T_2_(G)
Aminopterin	424.80	404.84	.4937	202.4217	5638.8333	.2174
Convolutamide A	334.18	326.50	.4434	163.2518	4312.0000	.0843
Convolutamydine A	113.89	103.34	1.3709	51.6685	768.5972	.4191
Daunorubicin	635.63	597.36	.4936	298.6796	10270.3333	.2072
Minocycline	453.42	421.33	.4965	210.6655	6729.2500	.2572
Podophyllotoxin	242.93	232.44	.6013	116.2185	3116.0000	.2794
Caulibugulone E	78.76	60.41	.8717	37.5380	445.1110	.3654
PerfragilinA	116.41	107.78	1.2321	53.8880	820.7780	.5396
Melatonin	120.81	115.78	1.0205	57.8890	852.8890	.4552
Tambjamine k	154.23	155.50	.5086	74.3290	1243.3890	.2350
Amathaspiramide E	180.71	165.53	.8283	85.2550	1590.0000	.3553
Aspidostomide E	269.73	256.91	.7857	128.4550	2788.4170	.3609

https://doi.org/10.6084/m9.figshare.27002857.v1

**Table 4:** The values of the temperature indices of the drugs.

Drugs	RPT(G)	SDT(G)	HT_1_(G)	HT_2_(G)	ST(G)
Aminopterin	2.6411	78.7029	.9286	.0016	81.5373
Convolutamide A	1.2272	59.9719	.3764	.0004	76.5136
Convolutamydine A	2.5316	50.3386	1.9278	.0129	30.3491
Daunorubicin	2.8443	103.8873	.8853	.0012	111.4924
Minocycline	2.9377	84.7168	1.0979	.0023	88.9976
Podophyllotoxin	2.7251	47.0252	1.1602	.0033	63.9580
Caulibugulone E	1.9689	35.5210	1.6025	.0137	23.5640
PerfragilinA	3.0151	46.3500	2.3113	.0198	30.9370
Melatonin	2.8059	42.1070	1.9309	.0133	32.1270
Tambjamine k	2.0497	45.9230	.9786	.0031	38.3870
Amathaspiramide E	2.6621	54.1030	1.5389	.0071	43.0260
Aspidostomide E	3.0966	65.8990	1.5074	.0055	59.6230

https://doi.org/10.6084/m9.figshare.27002857.v1

**Table 5:** Physicochemical properties of cancer drug.

Drugs	BP	EN	FP	MR	PSA	ST	MV	COM
Aminopterin	-	-	-	114.3	219	103.3	277.2	674
Convolutamide A	629.9	97.9	334.7	130.1	78	51.9	396	-
Convolutamydine A	504.9	81.6	259.2	68.2	66	59	190	363
Daunorubicin	770	117.6	419.5	130	186	87.4	339.4	960
Minocycline	803.3	122.5	439.6	116	165	90	294.6	971
Podophyllotoxin	597.9	93.6	210.2	104.3	93	52.8	302.4	583
Caulibugulone E	373	62	179.4	52.2	66	53.2	139.1	319
PerfragilinA	431.5	68.7	214.8	63.6	106	68.9	167.8	543
Melatonin	512.8	78.4	264	67.6	54	46.7	197.6	301
Tambjamine k	391.7	64.1	190.7	76.6	49	37	235.1	293
Amathaspiramide E	572.7	90.3	300.2	89.4	62	56.2	233.9	489
Aspidostomide E	798.8	116.2	436.9	116	59	71.4	262	572

https://doi.org/10.6084/m9.figshare.26985559.v1

### 3.2 Discussion and comparison of advanced machine learning models and linear models for QSPR analysis

The primary objective of this section is to conduct a QSPR analysis of various topological indices (TI) and examine their correlation with several physicochemical properties and activities of drugs. The drugs under investigation include Aminopterin, Convolutamide A, Convolutamydine A, Daunorubicin, Minocycline, Podophyllotoxin, Caulibugulone E, Perfragilin A, Melatonin, Tambjamine K, Amathaspiramide E, and Aspidostomide E. We assessed the effectiveness of these TI in predicting drug properties. We analyzed eight physicochemical properties: Boiling Point (BP), Enthalpy (EN), Flash Point (FP), Molar Refractivity (MR), Polar Surface Area (PSA), Surface Tension (ST), Molecular Volume (MV), and Complexity (COM), with values obtained from PubChem and Chemspider. Table 6 displays the correlation coefficients (r) between these physicochemical attributes and the degree-based topological indices. Tables 7–13 demonstrate that a linear QSPR model provides the best fit for predicting these properties. The values are normally distributed, and fifty-eight regression models were employed for data analysis. Notably, the PT(G), HT(G), ^m^T_3_(G), T_2_(G), and SDT(G) indices exhibit high correlations with COM, with R-values of 0.913, 0.905, 0.908, 0.915, and 0.905, respectively. Additionally, the ST) G (index shows a strong positive correlation with MR, with r = 0.924. In contrast, the RPT(G) topological index does not show a significant correlation with any physicochemical feature. The HT_1_(G) and T_2_(G) indices have a significant inverse correlation with MR and MV. The HT_2_(G) index is identified as the best predictor for BP, EN, MR, and MV, demonstrating an inverse correlation.

**Table 6:** Correlation coefficients of physical properties of drugs.

Drugs	BP	EN	FP	MR	PSA	ST	MV	COM
PT(G)	0.816	0.839	0.784	0.87	0.807	0.735	0.768	0.913
FT(G)	-0.467	-0.484	-0.385	-0.742	-0.444	-0.262	-0.765	-0.54
HT(G)	0.815	0.837	0.781	0.882	0.798	0.721	0.787	0.905
^m^T_3_(G)	0.815	0.838	0.781	0.878	0.802	0.726	0.781	0.908
T_2_(G)	-0.408	-0.444	-0.355	-0.758	-0.365	-0.178	-0.85	-0.506
^m^T_2_(G)	0.774	0.802	0.738	0.829	**0.808**	0.716	0.741	0.915
RPT(G)	0.369	0.341	0.318	-0.005	0.291	0.471	-0.253	0.574
HT_2_(G)	-0.626	-0.65	-0.531	-0.854	-0.413	-0.292	-0.863	-0.512
HT_1_(G)	-0.424	-0.455	-0.362	-0.772	-0.368	-0.178	-0.859	-0.512
ST(G)	**0.836**	0.86	0.767	**0.924**	0.764	0.682	**0.848**	**0.921**
SDT(G)	0.835	**0.854**	**0.857**	0.798	0.802	**0.808**	0.64	0.905

https://doi.org/10.6084/m9.figshare.26988049.v1

**Table 7:** Statistical metrics for the linear QSPR model applied to PT (G).

Physical Properties	N	A	b	R	R^2^	SE	F	P	Indicator
BP	11	.748	396.850	.816	.666	52.158	17.908	.000	significant
EN	11	.106	64.216	.839	.705	6.753	21.467	.000	significant
FP	11	.453	184.053	.784	.615	35.291	14.360	.001	significant
MR	12	.141	57.416	**.870**	**.756**	7.768	31.028	.000	significant
PSA	12	.271	29.617	.807	.651	19.323	18.668	.002	significant
ST	12	.085	42.638	.735	.540	7.654	11.731	.000	significant
MV	12	.333	166.264	.768	.589	27.034	14.356	.000	significant
COM	11	1.237	237.722	.913	.834	56.233	45.123	.002	significant

The significance of bold numbers denote highest correlation value

https://doi.org/10.6084/m9.figshare.26990038.v1

**Table 8:** Statistical metrics for the linear QSPR model applied to HT (G).

Physical Properties	N	A	b	R	R^2^	SE	F	P	Indicator
BP	11	.788	398.336	.815	.664	52.025	17.786	.000	significant
EN	11	.112	64.457	.837	.701	6.758	21.133	.000	significant
FP	11	.476	185.297	.781	.610	35.358	14.048	.001	significant
MR	12	.150	57.178	**.882**	**.777**	7.384	34.931	.000	significant
PSA	12	.282	30.878	.798	.637	19.599	17.577	.002	significant
ST	12	.088	43.220	.721	.519	7.781	10.809	.000	significant
MV	12	.359	164.758	.787	.619	25.904	16.252	.000	significant
COM	11	1.295	243.005	.905	.820	58.209	40.922	.002	significant

The significance of bold numbers denote highest correlation value

https://doi.org/10.6084/m9.figshare.26990662.v1

**Table 9:** Statistical metrics for the linear QSPR model applied to SDT (G).

Physical Properties	N	A	b	R	R^2^	SE	F	P	Indicator
BP	11	6.469	206.676	.835	.697	86.617	20.724	.001	significant
EN	11	.912	37.551	.854	.730	11.271	24.325	.009	significant
FP	11	4.187	53.365	**.857**	**.735**	51.095	24.950	.001	significant
MR	12	1.091	29.064	.798	.637	16.308	17.554	.002	significant
PSA	12	2.279	-35.475	.802	.643	33.620	18.029	.002	significant
ST	12	.792	17.651	.808	.653	11.432	18.829	.001	significant
MV	12	2.345	113.318	.640	.409	55.790	6.927	.025	significant
COM	11	10.271	-59.578	.905	.819	101.305	40.625	.000	significant

The significance of bold numbers denote highest correlation value

https://doi.org/10.6084/m9.figshare.26995264.v1

**Table 10:** Statistical metrics for the linear QSPR model applied to ^m^ T_3_ (G).

Physical Properties	N	A	b	R	R^2^	SE	F	P	Indicator
BP	11	1.589	395.950	.815	.664	52.542	17.751	.000	significant
EN	11	.225	64.102	.838	.702	6.816	21.176	.000	significant
FP	11	.961	183.747	.781	.610	35.651	14.094	.001	significant
MR	12	.301	56.854	**.878**	**.772**	7.547	33.795	.000	significant
PSA	12	.572	29.724	.802	.643	19.634	17.972	.002	significant
ST	12	.179	42.788	.726	.527	7.788	11.144	.000	significant
MV	12	.718	164.329	.781	.610	26.457	15.621	.000	significant
COM	11	2.618	238.144	.908	.824	57.996	42.193	.003	significant

The significance of bold numbers denote highest correlation value

https://doi.org/10.6084/m9.figshare.26995768.v1

**Table 11:** Statistical metrics for the linear QSPR model applied to ^m^T_2_ (G).

Physical Properties	N	A	b	R	R^2^	SE	F	P	Indicator
BP	11	.040	462.043	.774	.599	45.232	13.450	.000	significant
EN	11	.006	73.347	.802	.643	5.884	16.184	.000	significant
FP	11	.024	224.106	.738	.545	30.383	10.776	.000	significant
MR	12	.008	69.634	**.829**	**.688**	6.996	22.044	.000	significant
PSA	12	.015	50.813	.808	.654	15.325	18.874	.008	significant
ST	12	.005	49.727	.716	.512	6.273	10.494	.000	significant
MV	12	.018	194.512	.741	.549	22.557	12.162	.000	significant
COM	11	.070	333.617	.915	.838	44.485	46.431	.000	significant

The significance of bold numbers denote highest correlation value

https://doi.org/10.6084/m9.figshare.26996149.v1

**Table 12:** Statistical metrics for the linear QSPR model applied to HT_2_ (G).

Physical Properties	N	A	b	R	R^2^	SE	F	P	Indicator
BP	11	-15380.93	696.092	.626	.392	61.777	5.804	.000	significant
EN	11	-2199.264	106.779	.650	.422	8.297	6.578	.000	significant
MR	12	-3735.217	120.232	**.854**	**.730**	6.662	27.046	.000	significant
MV	12	-10116.120	323.901	.863	.745	17.370	29.183	.000	significant

The significance of bold numbers denote highest correlation value

https://doi.org/10.6084/m9.figshare.26996587.v1

**Table 13:** Statistical metrics for the linear QSPR model applied to FT (G).

Physical Properties	N	A	b	R	R^2^	SE	F	P	Indicator
MR	12	-65.545	143.984	.742	.550	15.348	12.239	.000	significant
MV	12	-181.194	391.033	**.765**	**.585**	39.540	14.093	.000	significant

The significance of bold numbers denote highest correlation value

https://doi.org/10.6084/m9.figshare.26997142.v1

Advanced machine learning models, including **SVR, Random Forest**, and **Linear Regression** (a traditional model), were employed for the analysis. The findings revealed the following key observations:

- SVR and Linear Regression models exhibited superior performance in predicting physicochemical properties, achieving correlation coefficients (r) above 0.9 for most properties. These results underscore the high predictive power of advanced machine learning techniques in QSPR analysis (Vapnik, 1995; Seber & Lee, 2003).
- The Random Forest model also showed acceptable performance. Although its accuracy was slightly lower than that of the tuned SVR and Linear Regression models, it provided valuable insights into the relationships between topological indices and drug properties (Breiman, 2001).
- In contrast, the SVR model demonstrated weaker performance, with lower correlation coefficients, highlighting the necessity of parameter optimization for achieving accurate predictions (Vapnik, 1995).

**Fig 3:**
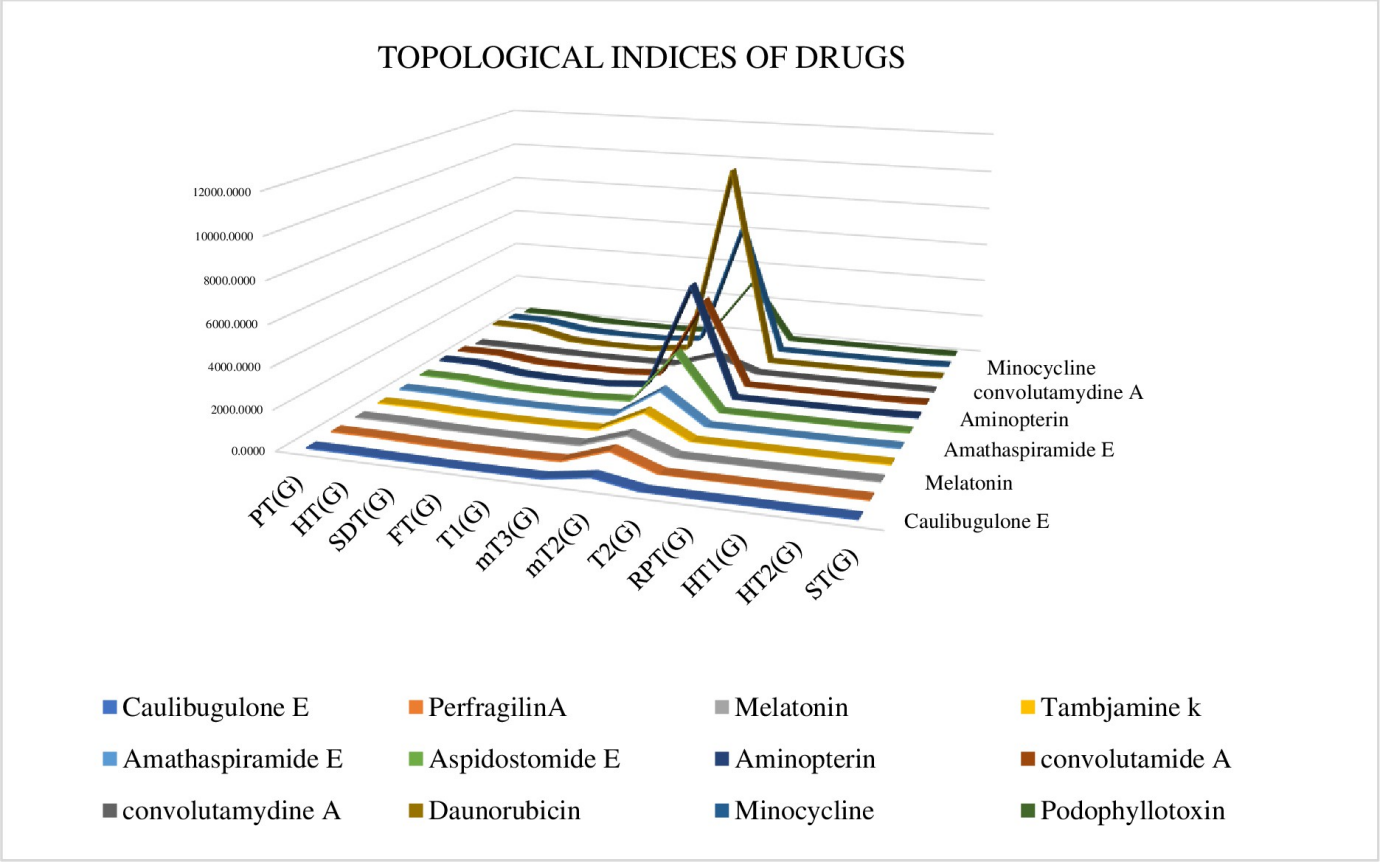
Two-dimensional (2D) graph illustrating the relationship between drugs and their topological indices. https://doi.org/10.6084/m9.figshare.26983915.v.

**Fig 4:**
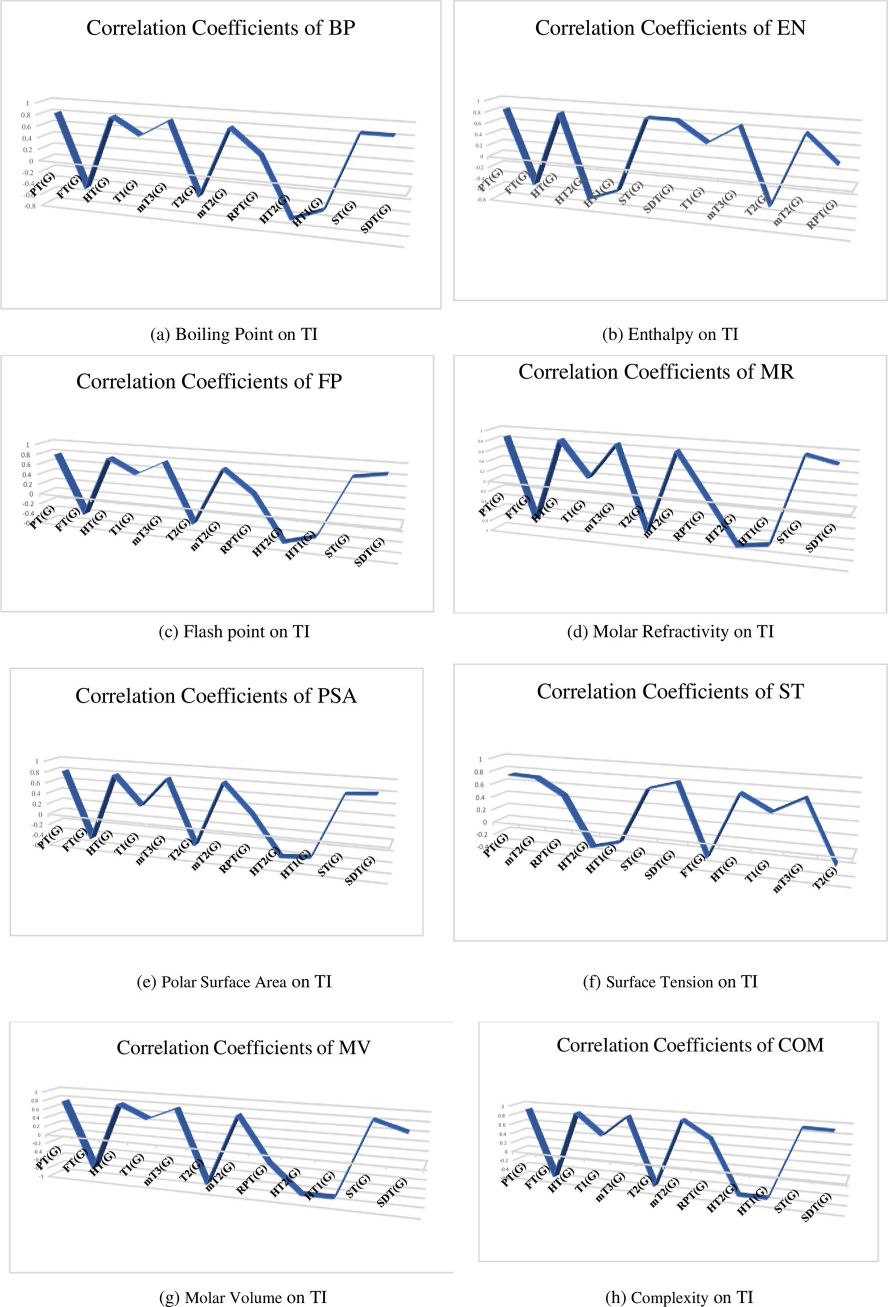
Physicochemical properties with topological indices. https://doi.org/10.6084/m9.figshare.26988649.v1.

Fig 3 provides a graphical representation of the correlations between TI and physicochemical properties. Fig 4 illustrates the relationship between TI and the physical properties of the drugs studied.

### 3.3 QSPR analysis

Building upon the temperature indices computed in Section 2, this section aims to develop a linear regression model. This model will be used to elucidate the relationships between the temperature indices and the physical and chemical properties of the drugs.


P=B+A(TI) (13)

Where:

- P: Represents the Anxiety drug property (dependent variable)
- B: Constant term (y-intercept)
- A: Regression coefficient
- TI: Topological index (independent variable)

Eq (13) represents the formulated linear regression model. In this equation, "P" denotes a specific property of an anxiety drug that we aim to predict or analyze. "B" is the constant term, and "A" is the regression coefficient, which indicates the change in "P" associated with a unit increase in the topological index. The analysis was performed using SPSS software to develop linear models for eight specific properties of cancer drugs across twelve different drugs. These models are based on the eleven topological indices computed earlier. The following section will present the various linear models tailored to each of the eight drug properties, using Eq (13) as the general framework.

### 3.4 Linear regression models

In this section, the linear regression models for topological indices (TI) are discussed using Eq (13). Tables 7–13 present the parameters and QSPR models associated with these TI. The following linear models for temperature indices are derived based on Eq (13):

**1. Product connectivity temperature index [PT (G)]**

BP = 396.850+0.748 [PT (G)], EN = 64.216+0.106 [PT (G)], FP = 184.053+0.453 [PT (G)]

MR = 57.416+0.141 [PT (G)], PSA = 29.617+0.271 [PT (G)], ST = 42.638+0.085 [PT (G)]

MV = 166.264+0.333 [PT (G)], COM = 237.722+1.237 [PT (G)]

**2. Harmonic temperature index [HT (G)]**

BP = 398.336+0.788 [HT (G)], EN = 64.457+0.112 [HT (G)], FP = 185.297+0.476 [HT (G)]

MR = 57.178+0.150 [HT (G)], PSA = 30.878+0.282 [HT (G)], ST = 43.220+0.088 [HT (G)]

MV = 164.758+0.359 [HT (G)], COM = 243.005+1.295 [HT (G)]

**3. Symmetric division temperature index [SDT (G)]**

BP = 186.910+6.469 [SDT (G)], EN = 37.003+.912 [SDT (G)], FP = 66.890+4.187 [SDT (G)]

MR = 26.265+1.091 [SDT (G)], PSA = -45232.412+2.279 [SDT (G)], ST = 10.415+0.792 [SDT (G)]

MV = 17.235+2.345 [SDT (G)], COM = 0.494+10.271 [SDT (G)]

**4. Modified third temperature index [^m^T_3_ (G)]**

BP = 395.950+1.589 [^m^T_3_ (G)], EN = 64.102+0.225 [^m^T_3_ (G)], FP = 183.747+0.961 [^m^T_3_ (G)]

MR = 56.854+0.301 [^m^T_3_ (G)], PSA = 29.724+0.572 [^m^T_3_ (G)], ST = 42.788+0.179 [^m^T_3_ (G)]

MV = 164.329+0.718 [^m^T_3_ (G)], COM = 238.144+2.618 [^m^T_3_ (G)]

**5. Modified second temperature index [^m^T_2_ (G)]**

BP = 462.043+0.040 [^m^T_2_ (G)], EN = 73.347+0.006 [^m^T_2_ (G)], FP = 224.106+0.024 [^m^T_2_ (G)]

MR = 69.634+0.008 [^m^T_2_ (G)], PSA = 50.813+0.015 [^m^T_2_ (G)], ST = 49.727+0.005 [^m^T_2_ (G)]

MV = 194.512+0.018 [^m^T_2_ (G)], COM = 333.617+0.070 [^m^T_2_ (G)]

**6. Second hyper temperature indices [HT_2_ (G)]**

BP = 696.092–15380.93 [HT_2_ (G)], EN = 106.779–2199.264 [HT_2_ (G)]

MR = 120.232–3735.217 [HT_2_ (G)], MV = 323.901–10116.120 [HT_2_ (G)]

**7. F-temperature index FT (G)**

MR = 120.232–3735.217 [FT (G)], MV = 323.901–10116.120 [FT (G)]

**8. First hyper temperature indices HT_1_ (G)**

MR = 120.232–3735.217 [HT_1_ (G)], MV = 323.901–10116.120 [HT_1_ (G)]

**9. Second temperature index T_2_ (G)**

MR = 120.232–3735.217 [T_2_ (G)], MV = 323.901–10116.120 [T_2_ (G)]

**10. Sum connectivity temperature index [ST (G)]**

BP = 327.804+4.642 [ST (G)], EN = 54.435+.658 [ST (G)], FP = 149.323+2.682 [ST (G)]

MR = 42.390+.911 [ST (G)], PSA = 11.502+1.565 [ST (G)], ST = 37.512+.481 [ST (G)]

MV = 125.946+2.239 [ST (G)], COM = 127.395+7.726 [ST (G)]

## 4 Machine learning models for predictive analysis

In this study, machine learning models were employed to predict the physicochemical properties of drugs used in the treatment of Cancer. The primary goal was to assess the potential of these models in identifying complex and nonlinear relationships between molecular structures and physicochemical properties. The use of machine learning methods in drug analysis offers the advantage of uncovering hidden patterns within the data that traditional methods may fail to identify (Vapnik, 1995).

### 4.1. Rationale for using machine learning models

Machine learning models are particularly suitable for capturing intricate, nonlinear relationships in large datasets. This is crucial for predicting drug properties, as these relationships are not always straightforward or linear. In this study, machine learning models were used to model these complex patterns and predict key physicochemical properties of drugs. These properties are vital for drug design, as they influence the drug’s behavior, efficacy, and safety profile. Traditional statistical methods often fail to account for these complexities, making machine learning an ideal choice.

For this analysis, in addition to linear regression, two other machine learning methods were used, which are described below:

1. **Support Vector Regression (SVR):** This model is well-known for its effectiveness in handling nonlinear data.
2. **Random Forest:** A model based on an ensemble of decision trees, which aggregates the predictions of many trees to improve accuracy and reduce overfitting. Random Forest is particularly effective for regression tasks in complex datasets.

These models were employed to predict the following physicochemical properties of the drugs: BP, EN, FP, MR, PSA, ST, MV, COM.

### 4.2. Comparison of prediction and analysis of models

**Fig 5:**
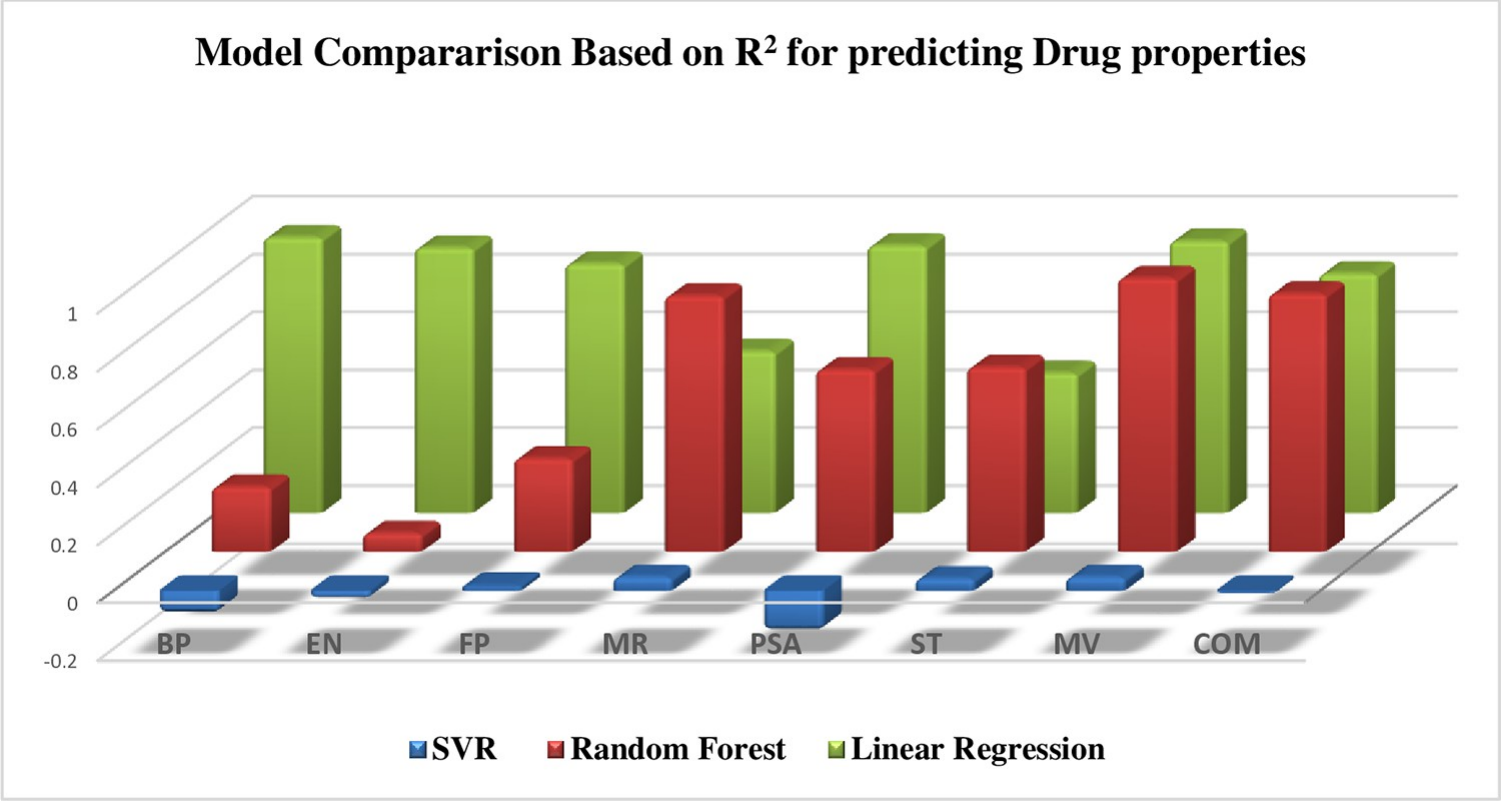
Comparison of machine learning models for predicting physicochemical properties of cancer drugs. https://figshare.com/articles/figure/_/28078031.

Linear regression performed the best, effectively capturing the relationships between the molecular structure of the drugs and their physicochemical properties. The SVR model also captured complex patterns but showed weaker results compared to linear regression. Random Forest performed the least well among the models. Tables 14–17 illustrate the predictions of physicochemical properties using different models, and the evaluation results are presented in Table 17 below and Fig 5.

**Table 14:** Prediction of physical and chemical properties using linear regression.

Drugs	BP	EN	FP	MR	PSA	ST	MV	COM
Aminopterin	502.9178	79.25277	262.1571	74.6904	62.24942	53.8369	174.9829	358.1457
Convolutamide A	594.2665	92.34224	266.1994	104.7399	99.77667	60.24787	276.7021	507.8311
Convolutamydine A	518.2478	81.03754	248.6585	67.98445	62.77917	54.50266	193.9989	370.6587
Daunorubicin	637.7867	98.20174	296.6422	129.8791	67.19081	52.72902	388.7722	79.93658
Minocycline	790.5111	117.5007	433.1623	112.5844	71.14037	68.90717	286.986	557.8229
Podophyllotoxin	813.7398	120.9429	420.0798	119.6955	147.3117	90.67948	298.7468	943.1044
Caulibugulone E	434.6103	67.74868	227.1406	61.39677	89.57761	62.5712	159.6744	642.1132
PerfragilinA	761.5278	118.4367	439.903	127.4511	197.2214	87.75863	344.8008	963.783
Melatonin	368.4921	63.03676	164.3571	49.57837	75.75286	50.06707	164.2359	188.6043
Tambjamine k	580.3727	90.93451	274.7751	85.99571	75.33736	58.83437	232.2445	390.0584
Amathaspiramide E	360.9571	71.81093	182.5549	72.85438	83.39079	45.07873	227.6464	104.2794
Aspidostomide E	-158.097	30.76467	134.8301	54.24999	234.2071	58.70372	308.8265	919.6087

https://doi.org/10.6084/m9.figshare.28077872

**Table 15:** Prediction of physical and chemical properties using SVR.

Drugs	BP	EN	FP	MR	PSA	ST	MV	COM
Aminopterin	594.9537	89.85808	264	99.86427	76.13678	59.12679	257.6979	542.093
Convolutamide A	597.9	93.6	266.3524	104.3	77.69741	59.49674	263.0683	543.2356
Convolutamydine A	595.1516	89.95788	264.1292	99.9392	76.19004	59	257.831	542.0788
Daunorubicin	600.0783	95.1467	267.7187	104.8855	78	59.11968	263.5021	542.3162
Minocycline	600.6772	95.18554	268.4679	105.0437	77.56055	61.31214	262	544.5428
Podophyllotoxin	601.0291	95.79631	269.0508	105.6726	79.49784	62.45004	263.8814	545.5563
Caulibugulone E	594.9814	89.89589	263.7808	99.74355	76.94799	59.89127	257.9344	543
PerfragilinA	600.6081	95.10032	268.7645	105.7717	79.61789	62.34736	263.6153	545.4268
Melatonin	595.0813	89.90094	264.2213	100.0452	76.27914	58.60532	258.1575	541.581
Tambjamine k	597.4321	92.19833	265.4958	102.2551	76.70804	59.64387	260.2539	542.5644
Amathaspiramide E	596.6249	91.67012	265.0912	102.1404	76.90827	58.59904	261.6032	541.9419
Aspidostomide E	598.0908	93.07143	266.3837	102.6357	78.01914	60.49908	263.9434	543.6364

https://figshare.com/articles/figure/_/28077872

**Table 16:** Prediction of physical and chemical properties using random forest.

Drugs	BP	EN	FP	MR	PSA	ST	MV	COM
Aminopterin	498.258	78.071	245.284	66.457	61.03	52.371	187.585	352.7
Convolutamide A	605.34	96.981	275.488	108.319	82.47	57.973	303.846	433.21
Convolutamydine A	510.428	80.512	253.288	68.335	65.53	57.259	188.059	368.43
Daunorubicin	657.242	102.172	339.641	122.584	85.62	53.639	363.324	182.19
Minocycline	779.287	113.26	395.533	111.29	76.27	70.345	265.033	505.38
Podophyllotoxin	783.54	118.527	429.158	116.84	144.64	84.586	306.266	880.7
Caulibugulone E	436.613	70.488	222.204	62.825	94.06	66.31	171.933	527.79
PerfragilinA	760.978	115.907	415.27	125.081	164.51	81.319	339.038	860.44
Melatonin	402.455	65.285	196.446	59.96	69.09	53.67	155.673	316.86
Tambjamine k	614.766	91.909	269.536	104.316	70.37	59.516	230.103	387.24
Amathaspiramide E	515.395	82.127	249.194	92.626	79.59	52.602	250.153	297.37
Aspidostomide E	641.264	104.75	330.928	96.938	114.34	68.999	314.702	707.87

https://figshare.com/articles/figure/_/28078031

**Table 17:** Evaluation of advanced machine learning models based on the coefficient of determination (R^2^) for predicting physicochemical properties of drugs.

Model	BP	EN	FP	MR	PSA	ST	MV	COM
**SVR**	-0.08055	-0.02977	0.017391	0.046939	-0.14016	0.041684	0.046667	-0.01107
**Random Forest**	0.224961	0.064874	0.322626	0.885989	0.630048	0.638303	0.946325	0.892501
**Linear Regression**	**0.952123**	**0.91647**	0.860965	0.558735	**0.923733**	0.482884	**0.939592**	0.827118

https://figshare.com/articles/figure/_/28078031

The linear regression model performed well in predicting most physical and chemical properties such as BP, EN, MR, and MV, with its predictions closely matching the actual values. Overall, the model is effective in modeling linear relationships.

The SVR model performed relatively well in predicting most physical and chemical properties, with predictions for BP**,** EN**,** MR, and MV being close to the actual values. Although the model showed reasonable accuracy for most properties, there were some discrepancies, especially for COM**.** Overall, the SVR model was effective in capturing complex, non-linear relationships in the data, but linear regression performed better in providing more accurate predictions.

The Random Forest model showed acceptable results in predicting the physical and chemical properties of the drugs, but compared to the Linear Regression and SVR models, its accuracy was lower in some predictions. For instance, for properties like BP and EN, there were notable discrepancies between the predicted values and the actual values, indicating lower precision in these cases. Therefore, it can be concluded that the Linear Regression and SVR models performed better in most cases, with their predictions being closer to the actual values.

As depicted in Fig 5, Linear regression demonstrated the best performance overall. Random Forest excelled in predicting non-linear relationships in some cases but showed lower accuracy in others. The SVR model exhibited weak performance.

Based on the evaluation of machine learning models using the coefficient of determination (R²) for predicting the physicochemical properties of drugs, linear regression demonstrated the best performance, achieving the highest R² values for most properties such as BP (0.95), EN (0.91), and MV (0.93), indicating strong predictive accuracy. Random Forest provided valuable insights into complex, non-linear relationships, though its accuracy was slightly lower than that of linear regression. Finally, SVR performed poorly and provided less accurate results compared to the other two models. Therefore, linear regression can be considered the best model for predicting the physicochemical properties of drugs.

## 5 Conclusion

Table 6 and Fig 3 illustrate the correlation between the physical and chemical properties of anti-cancer drugs and the defined temperature indices.

1. The Polar Surface Area is best predicted by the modified second temperature index, with a correlation coefficient (r) of 0.808.
2. The Sum Connectivity temperature index is the most effective predictor for Boiling Point (r = 0.836) and Molar Volume (r = 0.848). It also exhibits the highest significant correlations with Molar Refractivity (r = 0.924) and Complexity (r = 0.921).
3. The Symmetric Division temperature index shows a positive correlation with Enthalpy of Vaporization (r = 0.854), Flash Point (r = 0.857), and Surface Tension (r = 0.808).

This analysis reveals a positive correlation between the physical and chemical properties of Cancer drugs and the temperature indices. Tables 7–13 and 18–20 present regression models for various physical and chemical properties. The results demonstrate that the regression coefficients (r) exceed 0.6, and the p-values are below 0.05, indicating that these predictors are reliable for linear regression. The equations are formulated based on criteria such as minimum standard error (SE), maximum R-squared (R²), and maximum F-statistic. Consequently, it can be concluded that all physical and chemical properties are highly significant. This underscores the potential value of these topological indices in QSPR analysis for Cancer drugs, as evidenced by the plotted regression lines. The study’s findings can be applied to the production, development, and enhancement of more effective Cancer drugs. The theoretical insights derived from this study are beneficial for the development of new cancer therapies. Our findings reveal a clear trend in examining drug structures and their physical characteristics. Ultimately, this research contributes to the efficient design of new drugs and the development of preventive measures for the diseases in question. The principles of QSPR and topological indices offer valuable new approaches for estimating properties related to specific diseases and drugs, as demonstrated by the conclusions of this study. Furthermore, when comparing the three methods, despite the simplicity of Linear Regression, it consistently showed the best performance in predicting the physical and chemical properties of cancer drugs, outperforming both the SVR and Random Forest models. This emphasizes the effectiveness of Linear Regression in capturing the relationships within the data.

**Table 18:** Statistical metrics for the linear QSPR model applied to HT_1_ (G).

Physical Properties	N	A	b	R	R^2^	SE	F	P	Indicator
MR	12	-39.078	146.930	.772	.597	14.753	14.788	.000	significant
MV	12	-116.486	410.627	**.859**	**.737**	31.925	28.061	.000	significant

The significance of bold numbers denote highest correlation value

https://doi.org/10.6084/m9.figshare.26997166.v1

**Table 19:** Statistical metrics for the linear QSPR model applied to T_2_ (G).

Physical Properties	N	A	b	R	R^2^	SE	F	P	Indicator
MR	12	-167.663	146.781	.758	.575	15.364	13.503	.000	significant
MV	12	-503.865	411.470	**.850**	**.722**	33.321	25.928	.000	significant

The significance of bold numbers denote highest correlation value

https://doi.org/10.6084/m9.figshare.26997214.v1

**Table 20:** Statistical metrics for the linear QSPR model applied to ST (G).

Physical Properties	N	A	b	R	R^2^	SE	F	P	Indicator
BP	11	4.642	327.804	.836	.700	61.623	20.968	.000	significant
EN	11	.658	54.435	**.860**	**.740**	7.893	25.674	.000	significant
FP	11	2.682	149.323	.767	.588	45.519	12.830	.010	significant
MR	12	.911	42.390	.924	.854	7.471	58.536	.000	significant
PSA	12	1.565	11.502	.764	.584	26.243	14.014	.004	significant
ST	12	.481	37.512	.682	.464	10.263	8.673	.004	significant
MV	12	2.239	125.946	.848	.718	27.831	25.508	.001	significant
COM	11	7.726	127.395	.921	.848	67.000	50.092	.000	significant

The significance of bold numbers denote highest correlation value

https://doi.org/10.6084/m9.figshare.26997193.v1

## References

[1] Ghorbani M, Hosseinzadeh MA. A new version of Zagreb indices. Filomat. 2012;26(1):93–100.

[2] Kulli VR, Pal M, Samanta S, Pal A. Handbook of Research of Advanced Applications of Graph Theory in Modern Society. Hershey, USA: Global; 2020.

[3] Ghods M, Ramezani Tousi J. Computing Revan Polynomials and Revan Indices of Copper (I) Oxide and Copper (II) Oxide. Communications in Combinatorics, Cryptography & Computer Science. 2021;1(1):50–8.

[4] Kosari S. On spectral radius and Zagreb Estrada index of graphs. Asian-European Journal of Mathematics. 2023;16(10):4167.

[5] Kosari S, Dehgardi N, Khan A. Lower bound on the KG-Sombor index. Communications in Combinatorics and Optimization. 2023;8(4):751–7.

[6] Ramezani Tousi J, Ghods M. Computing K Banhatti and K Hyper Banhatti Indices of Titania Nanotubes TiO_2_ [m, n]. Journal of Information and Optimization Sciences. 2023;44(2):207–16.

[7] Ramezani Tousi J, Ghods M. Investigating Banhatti indices on the molecular graph and the line graph of Glass with M-polynomial approach. Proyecciones Journal of Mathematics. 2024;43(1):199–219.

[8] Shi X, Kosari S, Hameed S, Shah AG, Ullah S. Application of connectivity index of cubic fuzzy graphs for identification of danger zones of tsunami threat. PLoS ONE. 2024;19(1):1–24. doi: 10.1371/journal.pone.0297197 38289906 PMC10826963

[9] Havare ÖÇ. Quantitative structure analysis of some molecules in drugs used in the treatment of COVID-19 with topological indices. Polycyclic Aromatic Compounds. 2022;42(8):5249–60.

[10] Huang L, Wang Y, Pattabiraman K, Danesh P, Siddiqui MK, Cancan M. Topological indices and QSPR modeling of new antiviral drugs for cancer treatment. Polycyclic Aromatic Compounds. 2023;43(9):8147–70.

[11] Breiman L. (2001). Random forests. *Machine Learning*, 45(1), 5–32. 10.1023/A:1010933404324.

[12] Vapnik V. N. **(**1995). *The nature of statistical learning theory*. Springer.

[13] Ghani MU, Sultan F, Tag El Din ESM, Khan AR, Liu JB, Cancan M. A Paradigmatic Approach to Find the Valency-Based K-Banhatti and Redefined Zagreb Entropy for Niobium Oxide and a Metal–Organic Framework. Molecules. 2022;27(20):6975. doi: 10.3390/molecules27206975 36296567 PMC9610924

[14] Fajtolowicz S. On conjectures of Graffitti. Discrete Mathematics. 1988;72(1):113–8.

[15] PubChem. PubChem: National Center for Biotechnology Information [Internet]. [cited 2024 Sep 11]. Available from: https://pubchem.ncbi.nlm.nih.gov/.

[16] Kulli VR. Computation of Some Temperature Indices of HC_5_C_5_ [p, q] Nanotubes. Annals of Pure and Applied Mathematics. 2019;20(2):69–74.

[17] Kulli VR. Inverse sum temperature index and multiplicative inverse sum temperature index of certain nanotubes. International Journal of Recent Scientific Research. 2021;12(01):40635–9.

[18] Husin MN, Khan AR, Awan NUH, Campena FJH, Tchier F, Hussain S. Multicriteria decision making attributes and estimation of physicochemical properties of kidney cancer drugs via topological descriptors. PLoS ONE. 2024;19(5): e0302276. doi: 10.1371/journal.pone.0302276 38713692 PMC11075897

[19] Jahanbani A, Khoeilar R, Cancan M. On the Temperature Indices of Molecular Structures of Some Networks. Journal of Mathematics. 2022;2022(1):1–7.

[20] Kansal N, Garg P, Singh O. Temperature-based topological indices and QSPR Analysis of COVID-19 Drugs. Polycyclic Aromatic Compounds. 2023;43(5):4148–69.

[21] Ramezani Tousi J, Ghods M. Some polynomials and degree-based topological indices of molecular graph and line graph of Titanium dioxide nanotubes. Journal of Information and Optimization Sciences. 2024;45(1):95–106.18.

[22] Zhang Y, Khalid A, Siddiqui MK, Rehman H, Ishtiaq M, Cancan M. On analysis of temperature based topological indices of some Covid-19 drugs. Polycyclic Aromatic Compounds. 2023;43(4):3810–26.

[23] ChemicalBook. ChemicalBook: Chemical Information [Internet]. [cited 2024 Sep 11]. Available from: https://www.chemicalbook.com/.

[24] ChemSpider. (2021). Search asd shace chemistry. Retrieved from http://www.chemspider.com/AboutUs.aspx.

