# Supplementary material for: Innovative approaches in QSPR modelling using topological indices for the development of cancer treatments
Source: PLoS One. 2025 Feb 21;20(2):e0317507. doi: 10.1371/journal.pone.0317507 (PMC11844895; doi:10.1371/journal.pone.0317507)
Supplement: Attachment — Submitted filename: Response to reviewers.pdf [file pone.0317507.s001.pdf]

PONE-D-24-27529
Innovative Approaches in QSPR Modelling Using Topological Indices for the Development of Cancer Treatments
PLOS ONE

Dear Esteemed Reviewers,

we would like to sincerely thank you for your valuable and constructive feedback. Following the receipt of the reviewers' comments, the revised manuscript has been updated according to the PLOS ONE journal's requirements, including proper file naming and content edits consistent with the journal's style. Furthermore, we utilized the code provided in the given link to ensure that the findings presented in the manuscript are accurately supported. In the revised version, the data set, which initially included 6 drugs and 6 indices, has been expanded to include 12 drugs and 11 indices. Additionally, the issue of text overlap has been completely resolved. The introduction has been thoroughly revised, and the methods and analyses of the research process have been clearly articulated. As a result, readers will gain a clear understanding of the nature of the paper from this abstract, and the relationship between indices and drugs has been analyzed with greater precision based on the comprehensive revisions made. Specifically, the number of indices has increased from 6 to 11, the number of drugs from 6 to 12, and the number of regression lines to 58. These changes allow for a more comprehensive analysis of the characteristics and properties of the data. Moreover, with the addition of new data, the correlation of regression lines has approached statistically significant levels. In this context, indices with higher correlations have been included in the paper, while indices with low or no correlation have been excluded. Only those indices that showed higher correlation according to the graphical models of drugs are presented. Based on the reviewers' comments, the molecular weight, which was one of the physicochemical properties, has been removed and replaced with complexity and molar volume, for which acceptable correlations were found. The paragraphs highlighted by the reviewers have been removed, and the topological indices are now expressed with mathematical symbols. Additionally, a reference from PubChem has been added to the references section. This paper addresses topics across chemistry, mathematics, and statistics, illustrating its interdisciplinary nature. To emphasize this interdisciplinary approach, it is noteworthy that drug structures were initially sourced from the Chemical Book and then converted into molecular graph models using ChemDraw software. Subsequently, mathematical methods were employed to count graph edges and identify relevant indices for analysis. Physical and chemical property data for the drugs were gathered from the PubChem website. Statistical methods (QSPR) were then applied to analyze the relationships between topological indices and these properties. The results demonstrate that quantitative structure–activity relationship (QSAR) modeling significantly aids in the analysis of molecular graphs and drug development. In molecular graphs, QSPR helps to improve drug design and optimization by identifying and analyzing key structural features affecting drug performance. This approach can be beneficial in developing new drugs with optimal properties, reducing costs, and time-consuming tests in the early stages of research and development. Overall, QSPR acts as a powerful predictive tool that facilitates drug design and clarifies complex relationships between molecular structure and drug properties through graphical models. In summary, the revised manuscript has seen an increase in the number of indices and drugs, and all indices have been thoroughly analyzed. Furthermore,

the highest correlations among these indices have been identified and highlighted, and we have made the most of the valuable feedback provided by the esteemed reviewers.

Sincerely,

PONE-D-24-27529
Innovative Approaches in QSPR Modelling Using Topological Indices for the Development of Cancer Treatments
PLOS ONE

Reviewer #1: In this paper, the authors conducted a QSPR analysis for drugs used in cancer treatment using temperature indices. This paper cannot be recommended for publication due to the following major flaws:
1. Introduction is over brief and not suitable for a research article.

Answer: The introduction of the article has been thoroughly revised, and the methods and analysis of the research process are clearly outlined. Consequently, readers will gain insight into the nature of the article by reading this abstract.

2. Novelty is completely lacking. It does not look like a research article, rather a mere class assignment of high school students.

Answer: According to the comprehensive revisions made in the article, the relationship between indicators and drugs has been analyzed more precisely. Specifically, the number of indicators increased from 6 to 11, the number of drugs increased from 6 to 12, and the number of regression lines increased to 58 according to the significance of the data. These changes allow for a more comprehensive analysis of features and characteristics. And with the inclusion of additional data, the correlation of the regression lines is closer to statistical significance.

3. The physical properties considered in the QSPR study are well known and already studied. Unfortunately the focus on already known correlations with physical properties (such as the molecular weight) presented in the current version of the manuscript is not attractive for the readership.

Answer: We removed molecular weight, a physicochemical property, based on the opinion of respected reviewers, and replaced it with complexity and molar volume, for which we found an acceptable correlation. Additionally, we excluded the DE feature due to its lack of correlation with all indicators.

4. The essential advance of the present work with respect to the state-of-the-art in the field is completely lacking in the manuscript. There is no emphasis on the reasons why this work is of interest for the broader, multidisciplinary readership of the journals, which spans over fields as diverse as chemistry, physics, biology, mathematics, engineering and materials science. There are no any evidences about how this work advances the current body of knowledge.

Answer: This paper addresses topics across **chemistry**, **mathematics**, and **statistics**, illustrating its interdisciplinary nature. To emphasize this interdisciplinary approach, it is noteworthy that drug structures were initially sourced from the **Chemical Book** and then converted into molecular graph models using **ChemDraw** software. Subsequently, mathematical methods were employed to count graph edges and identify relevant indices for analysis. Physical and chemical property data for the drugs were gathered from the **PubChem** website. Statistical methods **(QSPR)** were then applied to analyze the relationships between topological indices and these properties. The results

demonstrate that quantitative structure–activity relationship (QSAR) modeling significantly aids in the analysis of molecular graphs and drug development. In molecular graphs, QSPR helps to improve drug design and optimization by identifying and analyzing key structural features affecting drug performance. This approach can be beneficial in developing new drugs with optimal properties, reducing costs, and time-consuming tests in the early stages of research and development. Overall, QSPR acts as a powerful predictive tool that facilitates drug design and clarifies complex relationships between molecular structure and drug properties through graphical models.

5. Sample size is a major factor in performing QSPR regression analysis, a sample of only 6 drugs is insufficient to derive a valid conclusion. Kindly refer https://doi.org/10.1016/j.comptc.2023.114108 and https://doi.org/10.1080/1062936X.2023.2239149, where the authors have performed regressions for more than 50 compounds. Further, the authors fail to test the regression model by comparing its results with the existing one, refer https://doi.org/10.1140/epjp/s13360-024-04939-0 , where the authors have performed a comparative analysis between the predicted values and the actual values.

Answer: In the revised article, the number of indicators was increased from 6 to 11, the number of drugs from 6 to 12, and the number of regression lines to 58. The AGT(G) index was removed because it did not show a significant correlation with the indicators. We introduced the indices HT(G), FT(G), T_2(G), RPT(G), HT1(G), and HT_2(G) due to their strong and significant correlations with the relevant properties, as presented in Tables 4 and 5.

6. discussion and analysis of results is just dropped in the manuscript. Saying that "a certain topological index has highest correlation among all other indices" without any further explanation or analysis is not useful or acceptable.

Answer: In the revised article, the number of indicators and drugs has been increased and all indicators have been comprehensively analyzed. In addition, according to the statistical results, the highest correlation among these indicators has been identified and highlighted.

7. Why have you restricted the study to these selective topological indices, given that there are many available, more than 3000?

Answer: We examined a larger number of topological indicators and those that have the highest correlation with respect to the molecular graph structure and physical and chemical properties are stated in the article. In contrast, we excluded other indices due to their low or no correlation and presented only the indices that showed higher correlations based on graphical models of drugs.

PONE-D-24-27529
Innovative Approaches in QSPR Modelling Using Topological Indices for the Development of Cancer Treatments
PLOS ONE

Reviewer #2: Similar molecules are available in the publication 'https://doi.org/10.1016/j.heliyon.2020.e04235'. However, since the authors' results are better than the above publication, I think it will make a significant contribution to the field of chemical graph theory.

Answer: We carefully reviewed the relevant paper and, based on the methods and results presented, increased the number of indices and drugs. These adjustments allowed us to achieve more acceptable statistical results and provide more accurate analyses.

1. A reference should be added for Table 4.

Answer: Correct, a reference from PubChem has been added to the references.

15. PubChem. PubChem: National Center for Biotechnology Information [Internet]. [cited 2024 Sep 11]. Available from: https://pubchem.ncbi.nlm.nih.gov/

2. Remove the following paragraph from the text:
"Using the definition 2 and Table 2, we can calculate the following topological indices for A:

- PT(A) index
- ST(A) index
- SDT(A) index
- mT3(A) index
- mT2(A) index
- AGT(A) index."

Answer: Editing and comments were deleted.

3. The results of Theorems 1 and topological index values of other molecules are shown in Table 5. Correct the sentence and add it under the proof of Theorem 1 together with Table 5.

4. Correct 'a' in tables with 'A'.

Answer: Edited and completed.

5. Write topological indexes in mathematical characters.

Answer: Edited and completed.
