# Supplementary material for: Innovative approaches in QSPR modelling using topological indices for the development of cancer treatments
Source: PLoS One. 2025 Feb 21;20(2):e0317507. doi: 10.1371/journal.pone.0317507 (PMC11844895; doi:10.1371/journal.pone.0317507)
Supplement: Attachment — Submitted filename: Response to Reviewers.docx [file pone.0317507.s002.docx]

PONE-D-24-27529R1

**Innovative Approaches in QSPR Modelling Using Topological Indices for the Development of Cancer Treatments.**

PLOS ONE

Response to Reviewers:

2. Is the manuscript technically sound, and do the data support the conclusions?

The manuscript must describe a technically sound piece of scientific research with data that supports the conclusions. Experiments must have been conducted rigorously, with appropriate controls, replication, and sample sizes. The conclusions must be drawn appropriately based on the data presented.

Reviewer 1: No

Reviewer 2: Yes

**Answer:**
Yes, the manuscript is technically sound, and the reasons are outlined below:

**1. Comprehensive Methodology:**
In this study, in addition to the **Linear Regression** model, two other models were also used for predicting molecular properties in the field of QSPR: **Support Vector Regression (SVR)** and **Random Forest**. All three models were carefully analyzed and compared based on performance metrics, such as the Coefficient of Determination (R²).

**Models:**

- **Linear Regression:**
  Linear Regression is a simple model that assumes a linear relationship between independent and dependent variables. It is widely used in prediction tasks and statistical analysis and serves as the baseline model for comparison with more complex models.
- **Support Vector Regression (SVR):**
  SVR is a machine learning technique inspired by **Support Vector Machines (SVM)** and specifically designed for regression tasks. It is highly effective in modeling complex, nonlinear relationships by using various kernels to map the data into higher-dimensional spaces.
- **Random Forest:**
  Random Forest is an ensemble model based on decision trees, where the predictions are aggregated from multiple trees to improve accuracy and reduce overfitting. It is particularly effective for complex datasets and nonlinear relationships.

**2. Results and Validity:**
The **Linear Regression model** achieved the highest R² values across all the tested properties, showing the best fit to the data compared to the more advanced models (SVR and Random Forest). The results demonstrated that Linear Regression not only provided accurate predictions but was also simpler and more interpretable, aligning with the study's objectives.

- **SVR and Random Forest** were explored for modeling complex nonlinear relationships but had consistently lower R² values than Linear Regression.
- Regression coefficients (r > 0.6) and p-values (< 0.05) validated the statistical significance of predictors in Linear Regression.
- Criteria such as minimum standard error (SE), maximum R², and maximum F-statistic were used to derive regression equations, ensuring methodological rigor.

**3. Scientific Soundness of Conclusions:**
The conclusions drawn in the manuscript are directly supported by the data:

- The results show that topological indices are reliable predictors in QSPR analysis for anticancer drugs.
- The physicochemical properties of anticancer drugs were found to be highly significant.

**4. Experimental Rigor:**
The study includes computational data collection, preprocessing, and appropriate controls to ensure the validity of the results. The detailed steps for each method are provided in the supplementary materials, enhancing reproducibility and transparency.

**5. Justification for Model Selection:**
Based on the results, Linear Regression was chosen as the final model because it achieved the best performance (highest R²) and simplicity, making it more suitable for the context of this research. This choice is justified by Table 20, which shows the comparative R² values for all models.

**Table 20: Evaluation of Advanced Machine Learning Models Based on the Coefficient of Determination (R^2^) for Predicting Physicochemical Properties of Drugs.**

| **Model** | **BP** | **EN** | **FP** | **MR** | **PSA** | **ST** | **MV** | **COM** |
| --- | --- | --- | --- | --- | --- | --- | --- | --- |
| **SVR** | -0.08055 | -0.02977 | 0.017391 | 0.046939 | -0.14016 | 0.041684 | 0.046667 | -0.01107 |
| **Random Forest** | 0.224961 | 0.064874 | 0.322626 | 0.885989 | 0.630048 | 0.638303 | 0.946325 | 0.892501 |
| **Linear Regression** | **0.952123** | **0.91647** | 0.860965 | 0.558735 | **0.923733** | 0.482884 | **0.939592** | 0.827118 |

In conclusion, the analyses and results presented in the manuscript are scientifically valid and rigorously derived. The data effectively support the conclusions, ensuring the manuscript’s technical soundness.

Note: Based on the attached codes, the details of the steps involved in the mentioned methods can be understood.

Attached:

<https://drive.google.com/file/d/1f5xGm25sB9NlUyrgqTA1PGBWbJ4FQ2w3/view?usp=sharing>

3. Has the statistical analysis been performed appropriately and rigorously?

Reviewer 1: No

Reviewer 2: Yes

Answer:

Yes, the statistical analysis has been performed appropriately and rigorously. The evaluation of the models' performance was based on the R² metric, which was chosen for its importance in assessing predictive and regression models. The R², or coefficient of determination, indicates the proportion of variance in the dependent variable (model output) that is explained by the independent variables (input features). The value of R² ranges from 0 to 1, with values closer to 1 indicating better model fit. A high R² value demonstrates the model's ability to effectively capture and predict relationships within the data. In this study, the Linear Regression model demonstrated the best performance with high R² values in predicting various physicochemical properties of drugs. Table 20 presents the evaluation of advanced machine learning models based on the coefficient of determination (R²) for predicting different drug properties. The Linear Regression model outperformed the other models in features such as BP (0.952123), EN (0.91647), FP (0.860965), PSA (0.923733), and others. In contrast, the SVR and Random Forest models performed significantly worse. Specifically, the SVR model showed lower R² values across all features, indicating its inability to accurately model the relationships between input and output variables. The Random Forest model performed better than SVR but still had significantly lower R² values compared to the Linear Regression model. All the data used in this research were collected from reliable computational sources and carefully preprocessed. Moreover, during the statistical analysis and model evaluation, all scientific standards, including experiment repetition and the use of appropriate controls, were strictly followed. The conclusions presented in this paper are entirely based on the data and analyses conducted, and the findings strongly support the results obtained from the Linear Regression model. Therefore, the selection of the Linear Regression model was not only based on its highest R² value but also on its simplicity and better interpretability.

4. Have the authors made all data underlying the findings in their manuscript fully available?

The [PLOS Data policy](http://track.editorialmanager.com/CL0/http:%2F%2Fwww.plosone.org%2Fstatic%2Fpolicies.action%23sharing/1/010f0193566d6adf-e28283a6-06d8-4473-9a91-d9f3f46fc3d0-000000/iAGXVLTMk3meJX-qHbOY6gtPZilkw5ywUmAMkXpVlU8=186) requires authors to make all data underlying the findings described in their manuscript fully available without restriction, with rare exception (please refer to the Data Availability Statement in the manuscript PDF file). The data should be provided as part of the manuscript or its supporting information, or deposited to a public repository. For example, in addition to summary statistics, the data points behind means, medians and variance measures should be available. If there are restrictions on publicly sharing data—e.g. participant privacy or use of data from a third party—those must be specified.

Reviewer 1: (No Response)

Reviewer 2: Yes

Answer:

Yes, all data related to the findings of the manuscript are fully available and have been included in the article. All requirements regarding data accessibility and sharing have been met in accordance with the PLOS Data policy. Additionally, all figures and tables from the manuscript, along with the underlying data, have been made publicly available on Figshare. Any restrictions, if applicable, are clearly stated in the Data Availability Statement.

6. Review Comments to the Author

Please use the space provided to explain your answers to the questions above. You may also include additional comments for the author, including concerns about dual publication, research ethics, or publication ethics. (Please upload your review as an attachment if it exceeds 20,000 characters)

Reviewer 1: The authors failed to address the concerns raised in my previous review. The revisions are unsatisfactory and does not meet the publication standards.

Reviewer 2: The authors have made the necessary corrections. It is suitable for publication in PLOS ONE journal.

Answer:

Response to Reviewer 1:

Thank you for your comments. We have made every effort to address the concerns raised in your previous review. In this revision, we have carefully addressed the issues you mentioned and worked to resolve them thoroughly. If there are still any unresolved concerns, we would appreciate any further guidance you can provide to help us improve the manuscript.

Response to Reviewer 2:

Thank you for your positive feedback. We are glad that the revisions have met your approval, and we hope that the manuscript now aligns with the standards of PLOS ONE. We look forward to your final decision
